# Crossed cerebellar diaschisis-related supratentorial hemodynamic and metabolic status measured by PET/MR in assessing postoperative prognosis in chronic ischemic cerebrovascular disease patients with bypass surgery

**DOI:** 10.1007/s12149-022-01766-0

**Published:** 2022-07-05

**Authors:** Bixiao Cui, Yi Shan, Tianhao Zhang, Yan Ma, Bin Yang, Hongwei Yang, Liqun Jiao, Baoci Shan, Jie Lu

**Affiliations:** 1 Department of Radiology and Nuclear Medicine, Xuanwu Hospital, Capital Medical University, Beijing, China; 2 Beijing Key Laboratory of Magnetic Resonance Imaging and Brain Informatics, Beijing, China; 3 Institute of High Energy Physics, Beijing Engineering Research Center of Radiographic Techniques and Equipment, Chinese Academy of Sciences, Beijing, China; 4 School of Nuclear Science and Technology, University of Chinese Academy of Sciences, Beijing, China; 5 Department of Neurosurgery, Xuanwu Hospital, Capital Medical University, Beijing, China; 6 CAS Center for Excellence in Brain Science and Intelligence Technology, Shanghai, China

**Keywords:** Positron emission tomography/Magnetic resonance, Glucose metabolism, Cerebral blood flow, Surgery, Crossed cerebellar diaschisis

## Abstract

**Objective:**

Cerebral ischemic status is an indicator of bypass surgery. Both hemodynamics and glucose metabolism are significant factors for evaluating cerebral ischemic status. The occurrence of crossed cerebellar diaschisis (CCD) is influenced by the degree of supra-tentorial perfusion and glucose metabolism reduction. This study aimed to investigate the relationship between the CCD-related supra-tentorial blood flow and metabolic status before bypass surgery in patients with chronic and symptomatic ischemic cerebrovascular disease and the prognosis of surgery.

**Methods:**

Twenty-four participants with chronic ischemic cerebrovascular disease who underwent hybrid positron emission tomography (PET)/magnetic resonance (MR) before bypass surgery were included. Arterial spin labeling (ASL)-MR and FDG-PET were used to measure blood flow and metabolism, respectively. The PET images were able to distinguish CCD. The supratentorial asymmetry index (AI) and volume in the decreased blood flow region, decreased metabolism region and co-decreased region on the affected side, except for the infarct area, were respectively obtained before bypass surgery. The neurological status was determined using the National Institutes of Health Stroke Scale (NIHSS) and modified Rankin Scale (mRS) scores. Differences between CCD-positive (CCD +) and CCD-negative (CCD−) groups were investigated.

**Results:**

Fourteen (58%) of the 24 patients were diagnosed as CCD +. Before surgery, the NIHSS and mRS scores of the CCD + were significantly higher than those of the CCD− (1.0(1.0) vs. 0.0(1.0), *P* = 0.013; 1.0(1.5) vs. 0.0(1.5), *P* = 0.048). After the surgery, the NIHSS and mRS scores of the CCD + showed a significant decrease (0.0(1.0) to 0.0(0.0), *P* = 0.011; 0.0(0.5) to 0.0(0.0), *P* = 0.008). Significant differences were observed in the supra-tentorial decreased metabolism region (all *P*s ≤ 0.05) between the CCD + and CCD− groups, but no differences were observed in the preprocedural decreased supratentorial blood flow region (*P* > 0.05). The preprocedural NIHSS score was strongly correlated with the metabolism AI value in the decreased metabolism region (*r* = 0.621, *P* = 0.001) and the co-decreased region (*r* = 0.571, *P* = 0.004).

**Conclusions:**

Supratentorial blood flow and metabolism are important indicators of CCD. This study showed that CCD + patients benefited more from bypass surgery than CCD− patients. Staging based on CCD-related supra-tentorial blood flow and metabolic status by hybrid PET/MR may help to personalize treatment.

**Supplementary Information:**

The online version contains supplementary material available at 10.1007/s12149-022-01766-0.

## Introduction

Data from a nationwide community-based study indicated that ischemic stroke accounts for approximately 70% of all incident stroke cases [1]. For patients with a clinical presentation of ischemic attacks and ineffective medical therapy, superficial temporal artery-middle cerebral artery (STA-MCA) bypass surgery has demonstrated significant therapeutic efficacy in numerous studies [2, 3]. Several imaging studies have reported that the most crucial goal of surgical revascularization was the reduction of stroke occurrence and improvements in neurological outcomes [3, 4]. Cerebral ischemic status is an indicator of surgery. Hemodynamics and glucose metabolism are significant factors for evaluating cerebral ischemic status [5–8]. The Carotid Occlusion Surgery Study (COSS) in 2011 failed to show a clear benefit of bypass surgery in patients compared to conservative drug therapy [9]. Our previous study demonstrated that hybrid PET/MR can simultaneously measure changes in cerebral hemodynamic and metabolic patterns before and after STA-MCA bypass surgery [10]. However, we did not compare differences in the supra-tentorial hemodynamic and metabolic status before STA-MCA bypass surgery.

Crossed cerebellar diaschisis (CCD) has predominantly been studied through PET images. The incidence of CCD after supra-tentorial stroke may be 45% [11]. Baron et al. [12] first observed this phenomenon. Wang et al. [13] reported that CCD occurred due to a decrease in blood flow or metabolic activity of the related supra-tentorial region. Nocun et al. [14] showed that the degree of cerebral hypoperfusion was a determinant of CCD and correlated with the degree of cerebellar hypoperfusion in the chronic stage of stroke. Uchino et al. [15] reported that CCD may be an indicator of severe postoperative hyperperfusion in patients with chronic ischemic cerebrovascular disease. Patients with symptomatic ischemic cerebrovascular disease have a 2-year risk of subsequent ipsilateral ischemic stroke of 10–15%, but this risk increases to 25% for those with severe hemodynamic impairment [16]. The supra-tentorial hemodynamic and metabolic status plays an important role in personalizing treatment and selecting patients who will benefit the most from revascularization therapy. However, few studies describing the relationship between CCD-related supratentorial blood flow and metabolic status and bypass surgery outcomes are available.

The most important goal of surgical revascularization is to protect non-infarcted areas in which cerebral blood flow (CBF) or metabolism has decreased, as assessed by imaging. Magnetic resonance imaging (MRI) is an important diagnostic method for chronic ischemic cerebrovascular disease. Structural imaging first excludes infarcted areas. Arterial spin labeling (ASL) sequences can be used to evaluate potentially reversible tissue ischemia. ^18^F-FDG is widely used in the measurement of brain glucose metabolism. However, various physiological processes at different time points may change between imaging sessions. Hybrid positron emission tomography (PET)/magnetic resonance (MR) is an optimal approach to simultaneously acquiring the temporal and spatial matching of datasets that display different information about a disease process. Thus, this study aimed to investigate the relationship between the CCD-related supratentorial blood flow and metabolic status before bypass surgery and the prognosis of surgery.

## Materials and methods

### Patients

The study was approved by the Ethics Committee of our hospital. All subjects in the study provided written informed consent for the study protocols. From October 2017 to October 2019, 24 patients were enrolled. Twenty-one of these patients were male, and 3 patients were female. The mean ± SD patient age was 49.44 ± 9.03 years (range, 32–63 years), and all subjects met the following criteria: (1) clinical diagnosis of the unilateral internal carotid artery (ICA) or MCA steno-occlusive disease that was considered severe (>70%) based on digital subtraction angiography (DSA) [5, 17]; (2) no supra-tentorial infarction (abnormal signal on T2-FLAIR images) localized in the contralateral hemisphere; (3) no structural abnormality in the cerebellum or brain stem on MR imaging (MRI); (4) a history of transient ischemic attacks or complete stroke involving the relevant ICA or MCA territory and ineffective treatment with medication; and (5) PET/MR scans less than 1 month before surgery and successful postoperative vascular connection as determined by DSA. Clinical long-term follow-up was performed for all patients with a postoperative follow-up time of at least 1 year, with an average of 21.44 months [18]. The exclusion criteria included any contraindication for MRI and artifacts on MRI.

### PET/MR acquisition

Patients were evaluated before bypass surgery using a hybrid PET/MR system (Signa, GE Healthcare) with a 19-channel head and neck union coil. The glucose level of each patient was lower than 8 mmol/L. A dose of 3.7 MBq/kg ^18^F-FDG was administered intravenously after fasting for at least 6 h before PET imaging. Fifty minutes after injection, the patients were placed into the PET/MR scanner in a supine position and instructed to remain calm with their eyes closed. ^18^F-FDG PET images were acquired for 10 min. Attenuation correction was performed based on MR images, and the default attenuation correction sequence (Dixon MR sequences) was automatically prescribed and acquired as follows[19]: LAVA-Flex (GE Healthcare) axial acquisition, repetition time (TR) = 4 ms, echo time (TE) = 1.7 ms, slice thickness = 5.2 mm with a 2.6 mm overlap, 120 slices, pixel size = 1.95 × 2.93 mm, and acquisition time = 18 s. PET data were reconstructed into a 192 × 192 matrix, 35 cm field of view (FOV), and 2.78 mm slice thickness (voxel size 1.82 × 1.82 × 2.78 mm^3^) using a time-of-flight, point spread function, ordered subset expectation maximization (TOF-PSF-OSEM) algorithm with 8 iterations, 32 subsets, and a 3 mm Gaussian filter [10].

Simultaneous PET and MRI imaging data were acquired. The sequences obtained were conventional T2WI, T1WI, T2-fluid attenuated inversion recovery (FLAIR), and 3D ASL. The following parameters were used: T2-FLAIR with 32 slices, TR = 11,000 ms, TE = 144 ms, FOV = 240 × 240 mm, voxel size 0.47 × 0.47 × 4.00 mm^3^ and scan time = 176 s and 3D ASL with 36 slices, post labeling delay (PLD) = 2550 ms, TR = 5335 ms, TE = 10.7 ms, FOV = 240 × 240 mm, voxel size 1.88 × 1.88 × 4.00 mm^3^, and scan time = 310 s.

### Assessment of neurological and functional status

The National Institutes of Health Stroke Scale (NIHSS) score and the modified Rankin Scale (mRS) score were used to evaluate the neurological and functional status before the PET/MR examination as a baseline and at least at the 1-year follow-up [20, 21].

### Data processing and analysis

First, two experienced doctors from the Department of Radiology and Nuclear Medicine blinded to the ASL-CBF findings visually determined CCD based on ^18^F-FDG PET images following published criteria [22].

Second, two radiologists blinded to patient information measured the infarction area using T2-FLAIR images. For cases of disagreement, a consensus was reached in a separate session. All images were preprocessed using SPM8 (Wellcome Department of Clinical Neurology, London, UK) [10]. ^18^F-FDG-PET and ASL images were first spatially normalized to the MNI (Montreal Neurological Institute) space with a 3 × 3 × 3 mm^3^ resolution using affine transformation and subsequent nonlinear warping. The ^18^F-FDG-PET images were transformed into maps representing the radioactivity (kBq/ml), which was defined as the tissue concentration of radioactivity in each voxel normalized to the mean activity concentration. The CBF map was computed using the formula in reference [23]. All images were smoothed using an isotropic Gaussian kernel at full width at half maximum (FWHM) of 8 mm in all directions.

Parameters, including voxel-wise asymmetry index (AI) values for CBF and metabolism, were calculated after the exclusion of the infarct area from the entire supratentorial-affected side. The AI used to detect left–right asymmetry in the ^18^F-FDG PET and ASL-CBF data is based on the following equation:

$${\text{AI}} = ({\text{unaffected}}- {\text{affected}}) / {\text{unaffected}}\, { \times }\, 100\%$$

where unaffected stands for the CBF or radioactivity on the unaffected side, and affected stands for the CBF or radioactivity on the affected side. Abnormal asymmetry levels were defined as those greater than 10% [10, 22, 24].

We also defined the volume for which the voxel-wise AI value was greater than 10% as the abnormal volume, including the supratentorial decreased blood flow region, the supratentorial decreased metabolism region and the co-decreased region where both the CBF and metabolism decreased (Supplemental Fig. S1).

### Statistical analysis

The reliability of visual evaluation of CCD by the two doctors was determined using the intraclass correlation coefficient (ICC). ICC value of <0.20 indicated poor agreement, ICC value of 0.21–0.40 indicated fair agreement, ICC value of 0.41–0.60 indicated moderate agreement, ICC value of 0.61–0.80 indicated good agreement, and ICC value of >0.81 indicated excellent agreement.

We performed statistical analyses using SPSS Statistics 21. The normality of the distribution was evaluated using the Shapiro–Wilk test. Normally distributed continuous variables from the CCD-positive (CCD +) and CCD-negative (CCD−) groups were compared using a two-tailed, independent-samples Student’s *t*-test. Variables with a non-normal distribution were analyzed using the Mann–Whitney U-test. All normally distributed continuous variables are reported as medians (interquartile ranges). To identify a relationship between the parameters and neurological score using the NIHSS and mRS, we used Spearman rank-order correlation analysis. All tests were considered significant at the *P* < 0.05 level.

## Results

### Patient demographics and clinical characteristics

**Fig. 1:**
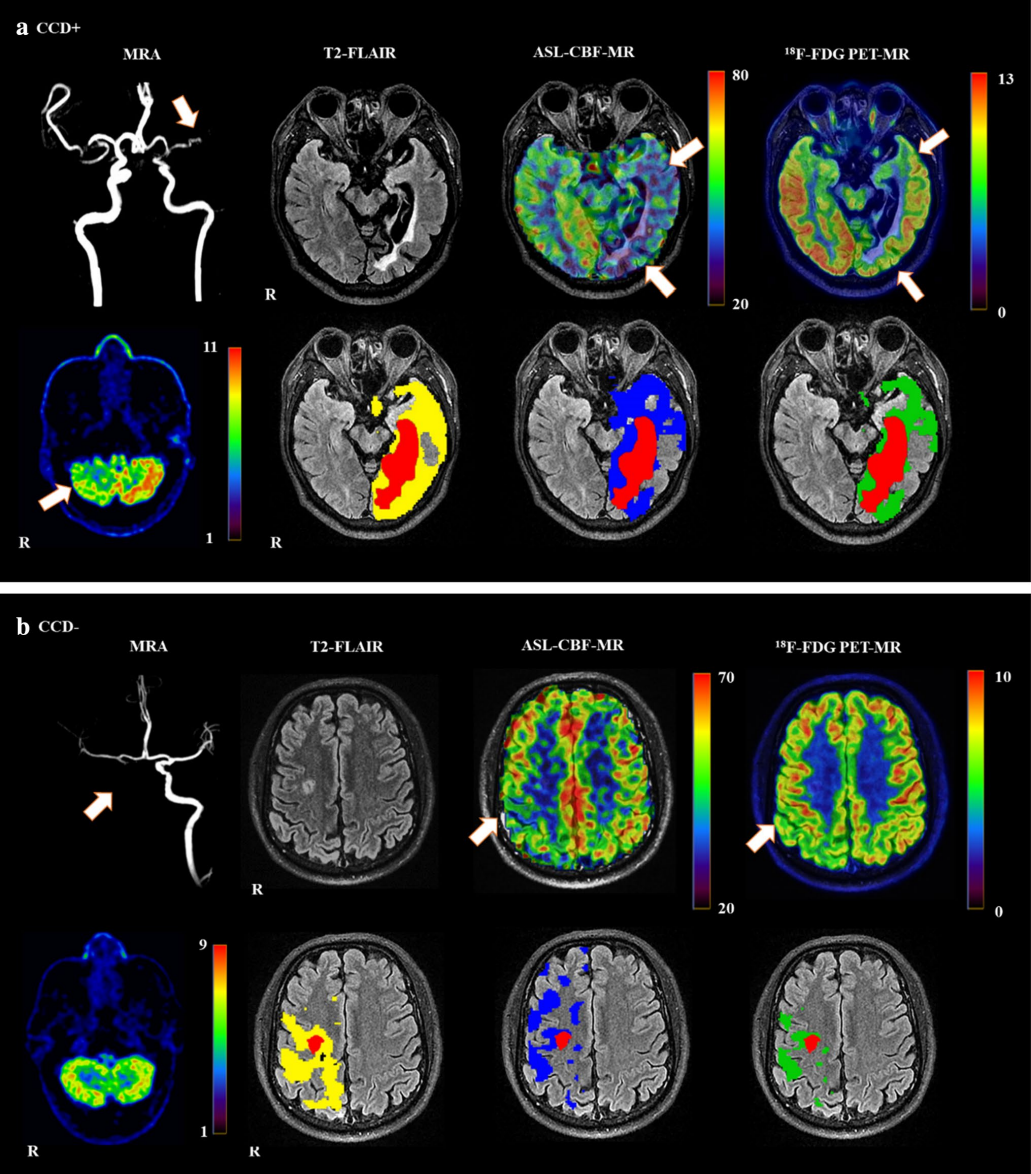
Example of a CCD-positive **a** and a CCD-negative **b** participant. **a** A 44-year-old man with ischemia in the left hemisphere due to left middle cerebral artery occlusion (white arrow). On arterial spin labeling (ASL)—cerebral blood flow (CBF) imaging and ^18^F-FDG positron emission tomography (PET), supratentorial hemodynamic and metabolic impairment may be seen in the left hemisphere (white arrows). Crossed cerebellar diaschisis (CCD) on ^18^F-FDG PET images shown of the cerebellum (white arrow). **b** A 62-year-old man with ischemia in the right paracentral hemisphere due to right internal carotid artery occlusion (white arrow). On ASL-CBF imaging and ^18^F-FDG PET, supratentorial metabolic and hemodynamic impairment is shown in the right hemisphere (white arrows), No CCD was observed on the cerebellar ^18^F-FDG PET image. Red—infarct zone in the supratentorial hemisphere. Yellow—decreased blood flow regions. Blue—decreased metabolism regions. Green—co-decreased regions

For CCD evaluation, excellent reliabilities were observed between the visual evaluation of CCD by the two doctors (ICC = 0.92; 95% confidence interval [CI] 0.82 to 0.96; *P* < 0.001). Fourteen (58%) of the 24 datasets were classified as CCD +. The CCD + and CCD− groups did not differ significantly in age or sex. Exemplary imaging illustrations of 1 CCD + and 1 CCD− participant are shown in Fig. 1. Detailed characteristics of the CCD + and CCD− patients are shown in Table 1. No differences in the blood glucose or injection activity were observed between these 2 groups.

**Table 1:** Clinical characteristics of the CCD + and CCD− groups

	CCD +	CCD−	*P* value
Age	46(12)	54(14)	0.300
Male (%)	13(93)	8(80)	0.550
Preprocedural, median (IQR)			
Blood glucose (mmol/L), median (IQR)	6.00(0.85)	6.00(0.85)	1.000
Injection activity (MBq)	302.11(53.00)	295.08 (87.67)	0.403
Size of infarction, median (IQR)	962.50(1820.00)	44.00(112.75)	<0.001
Baseline			
NIHSS score, median (IQR)	1.0(1.0)	0.0(1.0)	0.013
mRS score, median (IQR)	1.0(1.5)	0.0(1.5)	0.048
Follow-up			
NIHSS score, median (IQR)	0.0(1.0)	0.0(0.0)	0.172
mRS score, median (IQR)	0.0(0.5)	0.0(0.0)	0.625

*CCD* crossed cerebellar diaschisis; *CBF* cerebral blood flow; *AI* asymmetry index; *IQR* interquartile range

### Comparison of NIHSS and mRS between CCD-positive and CCD-negative groups

**Fig. 2:**
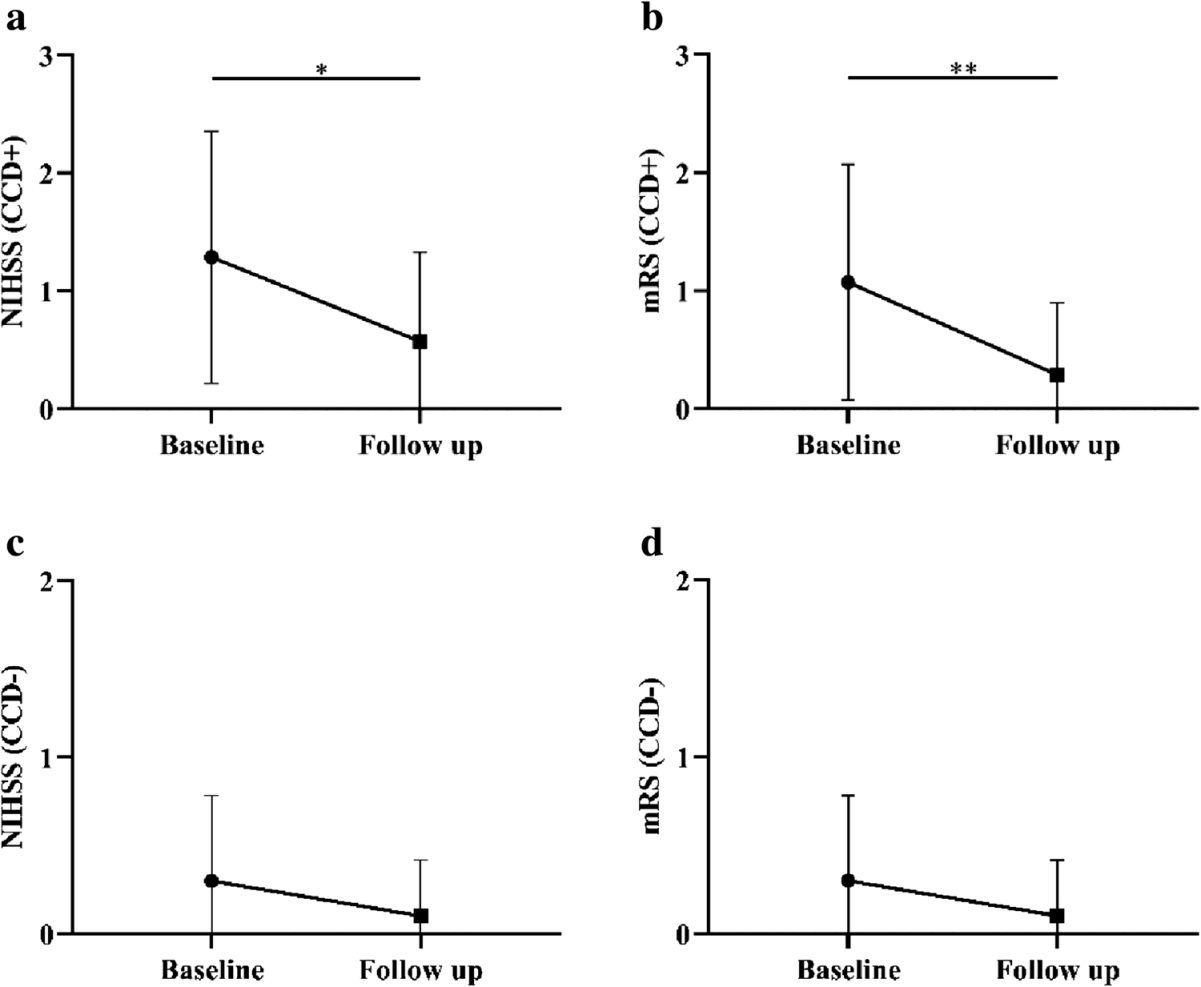
Comparison of baseline and follow-up neurological scores between the two groups. Both NIHSS and mRS scores decreased after surgery in the CCD-positive (CCD +) group (*P* = 0.011, *P* = 0.008, Fig. 2a, b). No significant change was found in the CCD-negative (CCD−) group (*P* = 0.157, *P* = 0.157, Fig. 2c, d). The dots and bars stand for mean with range. **P < 0.01, *P < 0.05

While all patients presented a favorable prognosis, the CCD + group showed a larger change in the NIHSS and mRS scores. Before surgery, the CCD + patients had higher preprocedural NIHSS and mRS scores than the CCD− patients (1.0(1.0) vs. 0.0(1.0), *P* = 0.013; 1.0(1.5) vs. 0.0(1.5), *P* = 0.048). The follow-up NIHSS and mRS scores at least 1 year after surgery were similar between the 2 groups (0.0(1.0) vs. 0.0(0.0), *P* = 0.172; 0.0(0.5) vs. 0.0(0.0), *P* = 0.625). The NIHSS and mRS scores decreased significantly after surgery in the CCD + group (0.0(1.0) to 0.0(0.0), *P* = 0.011; 0.0(0.5) to 0.0(0.0), *P* = 0.008, Fig. 2a, b), but no significant changes in the NIHSS and mRS scores were found in the CCD− patients (0.0(1.0) to 0.0(0.0), *P* = 0.157; 0.0(1.5) to 0.0(0.0), *P* = 0.157, Fig. 2c, d).

### Comparison of CBF and metabolism between CCD-positive and CCD-negative groups

**Fig. 3:**
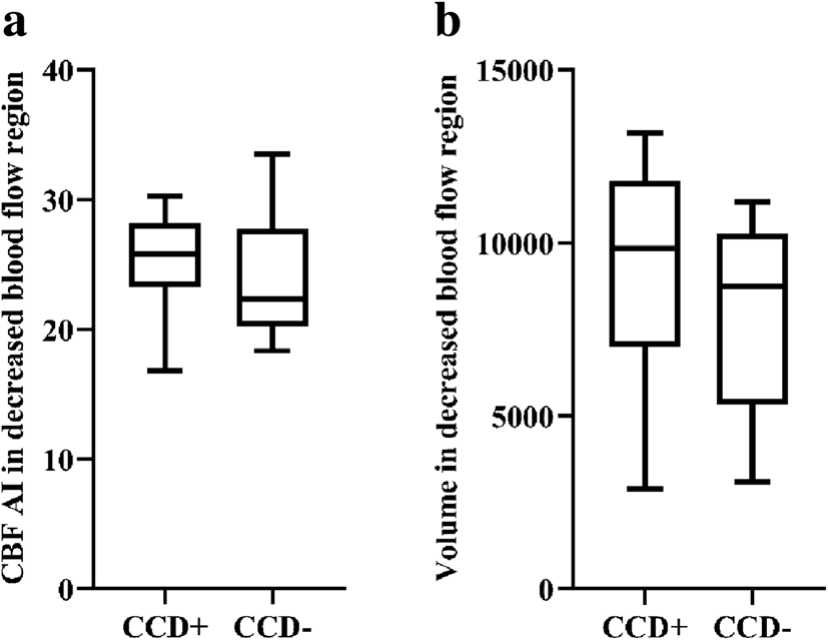
Comparison of parameters in the decreased blood flow region between the two groups. No difference in either CBF AI **a** or volume **b** was observed between the CCD-positive group and the CCD-negative group

**Fig. 4:**
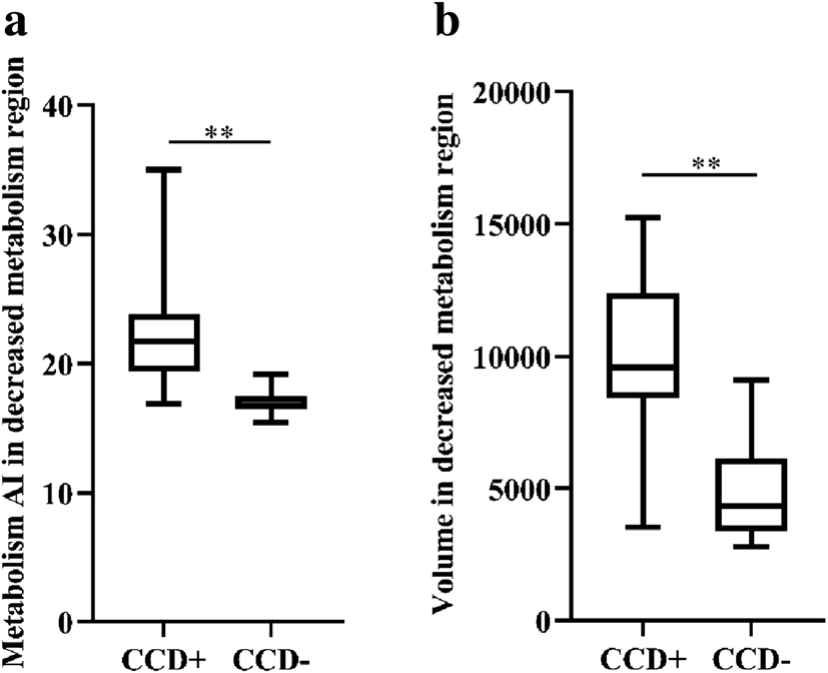
Comparison of regions with decreased metabolism between the two groups. CCD-positive patients showed significantly higher metabolism AI **a** and larger volume **b** than CCD-negative patients. **P < 0.01

**Fig. 5:**
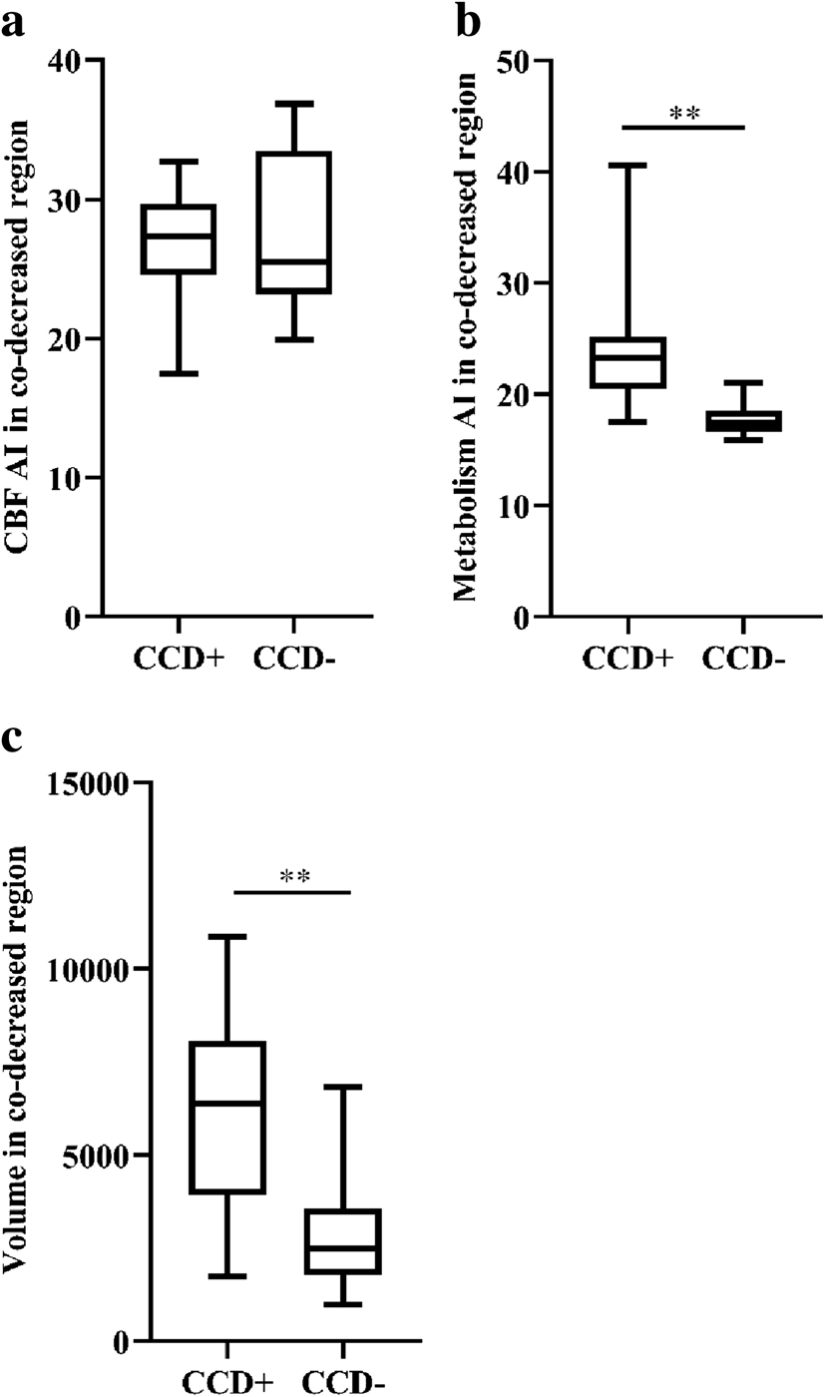
Comparison of parameters in a co-decreased region between the two groups. No difference in the CBF AI **a** between the CCD-positive group and the CCD-negative group. There was significantly higher metabolism AI **b** and larger volume **c** in CCD-positive patients. **P < 0.01

In the supratentorial decreased blood flow region, no statistically significant differences between the 2 groups were found in the preprocedural supratentorial CBF AI value or the volume (*P* > 0.05) (Fig. 3). In the supratentorial metabolism region, the CCD + patients showed significantly higher preprocedural supratentorial metabolism AI values and larger decreased preprocedural volume compared to the CCD− patients (CCD + vs. CCD−: 21.67 vs. 16.78, *P* = 0.001 and 9594.50 vs. 4353.00, *P* < 0.001) (Fig. 4). In the co-decreased regions which were defined as regions with decreased CBF and SUVR values, the metabolism AI and volume were also significantly higher in the CCD + patients (CCD + vs. CCD−: 23.03 vs. 17.43, *P* = 0.003; 6400.00 vs. 2491.00, *P* = 0.005). No significant difference in the CBF AI value was found in the co-decreased region between the two groups (*P* = 0.733) (Fig. 5). Detailed parameters of the CCD + and CCD− patients are shown in Table 2.

**Table 2:** Parameters in different regions between CCD + and CCD− patients

	CCD +	CCD−	*P* value
Decreased blood flow region			
CBF AI (%), median (IQR)	25.84(4.93)	22.37(7.50)	0.591
Volume, median (IQR)	9839.50(4794.00)	8729.00(4943.75)	0.364
Decreased metabolism region			
Metabolism AI (%), median (IQR)	21.67(4.37)	16.78(0.99)	0.001
Volume, median (IQR)	9594.50(3947.75)	4353.00(2732.00)	< 0.001
Co-decreased region			
CBF AI (%), median (IQR)	27.28(5.08)	25.50(10.28)	0.733
Metabolism AI (%), median (IQR)	23.03(4.70)	17.43(1.89)	0.003
Volume, median (IQR)	6400.00(4150.25)	2491.00(1814.00)	0.005

*CCD* crossed cerebellar diaschisis; *CBF* cerebral blood flow; *AI* asymmetry index; *IQR* interquartile range

### Correlations between NIHSS and metabolism characteristics

**Fig. 6:**
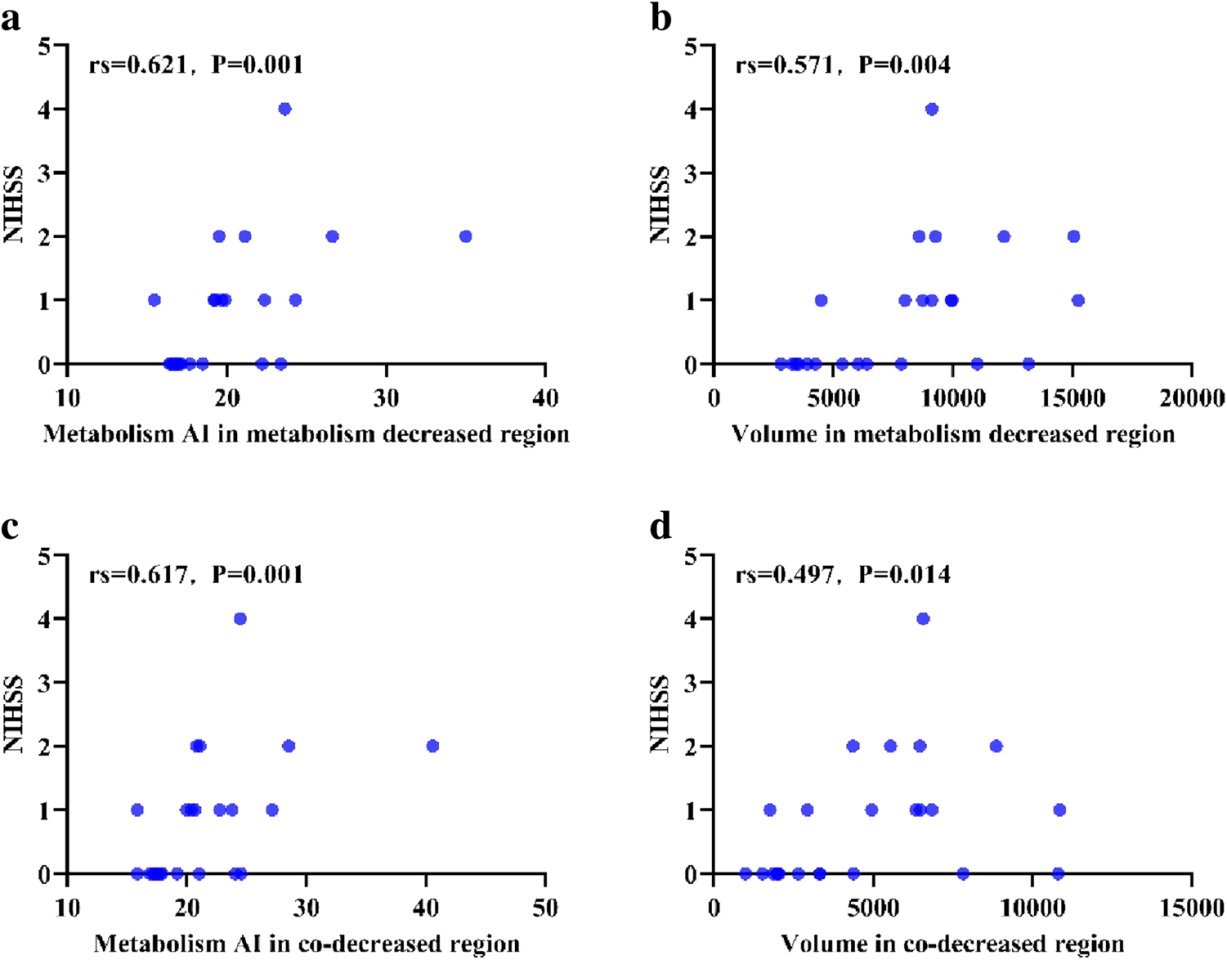
Correlations between neurological status and parameters. A significant correlation was observed between the NIHSS score and metabolism AI values in the decreased metabolism region (*r* = 0.621, *P* = 0.001) **a** and metabolism AI co-decreased region (*r* = 0.571, *P* = 0.004) **b** A more modest but significant correlation was found between the volume of the decreased metabolism region (*r* = 0.617, *P* = 0.001) **c** and the volume of the co-decreased region (*r* = 0.497, *P* = 0.014) **d**

The correlation analysis for these groups is shown in Fig. 6. Correlation analysis revealed that the preprocedural NIHSS score strongly correlated with the metabolism AI value in the decreased metabolism region (*r* = 0.621, *P* = 0.001) and the metabolism AI co-decreased region (*r* = 0.571, *P* = 0.004). A more modest but significant correlation was found between the volume of the decreased metabolism region (*r* = 0.617, *P* = 0.001) and the volume of the co-decreased region (*r* = 0.497, *P* = 0.014). No correlation was found between the CBF AI and clinical outcome measures preoperatively or at the last follow-up.

## Discussion

Cerebral perfusion and glucose metabolism are closely coupled to neural activity and are important markers of brain function. We retrospectively analyzed the ^18^F-FDG PET/MR results of 24 patients with the chronic ischemic cerebrovascular disease before bypass surgery and added CCD as a factor for investigating the CCD-related supratentorial hemodynamic and metabolic status before STA-MCA bypass surgery. Our results show that CCD may be an important factor in surgical selection in chronic symptomatic ischemic cerebrovascular disease.

Most previous reports mentioned CCD in patients with ischemic stroke [25, 26]. We found that 58% of the patients were CCD +, which is similar to the incidence reported by others [26, 27]. CCD is a common phenomenon when there is a supra-tentorial infarction. Although the size of the supra-tentorial infarctions in the CCD + group was larger in our study, Sommer et al.[28] reported that the occurrence of CCD is influenced by the degree of the reduction of supratentorial perfusions rather than the size of the infarction.

For the quantitative assessment of the level of functional impairment following a stroke, NIHSS and mRS scores are used in neurological practice. Our results show that the CCD + patients had higher preprocedural NIHSS and mRS scores. Szilagyi et al. [29] reported that patients with CCD had worse clinical stroke scale values than patients without CCD in subacute stroke. Kunz et al. [30] also reported that subacute neurological function and recovery were worse in patients with CCD than in patients without CCD. Therefore, the early identification of patients with CCD may support appropriate management strategies and avoid potentially harmful treatments.

Bypass surgery aims to protect brain tissue from infarction by increasing blood flow and reducing the stroke risk in patients [31]. Bai et al. [4] demonstrated that STA-MCA bypass is an effective and safe way to improve the regional ischemic status in ischemic cerebrovascular diseases. However, Powers et al. [9] reported that bypass surgery combined with medical therapy did not reduce the risk of recurrent stroke compared to medical therapy alone. One of the indications of this surgery is cerebral supratentorial ischemic status. The occurrence of CCD is related to supratentorial blood flow or metabolism. However, few studies describing the relationship between CCD-related supratentorial blood flow and the metabolic status and bypass surgery outcomes are available. We added CCD as a factor to compare the CCD-related supratentorial hemodynamic and metabolic status. Our results suggest that no significant differences were observed in the follow-up NIHSS and mRS scores between the two groups at 1 year. NIHSS and mRS scores decreased significantly after surgery in the CCD + group. These results demonstrated that surgical treatment was beneficial to the recovery of patients with CCD.

Theoretically, insufficient blood flow and metabolic impairment resulting from vascular occlusion after an ischemic stroke can cause tissue damage [32]. The supratentorial ischemic status is an important indicator of surgery. In our study, we focused on evaluating non-infarcted regions based on structural images because in the chronic ischemic stage, structural imaging is negative except for infarction areas. The risk increases to 25% for patients with severe hemodynamic impairment. Most studies documented that a region with decreased blood flow was important for patients with ischemic cerebrovascular disease after surgery [4, 33, 34]. However, few studies have focused on the CCD-related degree and size of supratentorial decreased blood flow regions and surgical outcomes. Although the supratentorial parameters of the decreased blood flow region in the CCD + group were higher than those in the CCD− group before surgery, no significant differences were observed in the supratentorial volume of the decreased blood flow region and CBF AI value between the two groups. Previous studies showed that ASL-MRI detected CCD with a relatively short PLD time (PLD = 1.5 s) [35, 36]. Long PLDs are required for ASL studies of supratentorial blood flow in the chronic stage [23]. The long PLD time selected by ASL and the supply of cerebral collateral circulation may have affected the results in this study [37]. We also found no correlation between the parameters in the decreased blood flow region and clinical outcome. Kunz et al. [30] demonstrated that the clinical score at the chronic stage was not available for all ischemic patients.

Maintaining normal brain homeostasis is a continuous energy-consuming process. In our previous study, 15 patients demonstrated improved glucose metabolism on ^18^F-FDG PET/MR after bypass surgery [10]. Yu et al. [8] performed a serial PET study in which ^18^F-FDG was used to investigate changes in glucose metabolism before and after bypass surgery and found that the uptake of ^18^F-FDG in the hypoperfusion region surrounding the ischemic core was a sensitive indicator for predicting ischemia. The present study retrospectively analyzed glucose metabolic alterations between CCD + and CCD− groups before bypass surgery. Significant differences were detected in the preprocedural parameters of the metabolism-decreased region. Correlation analysis showed that the preprocedural NIHSS score strongly correlated with metabolic parameters in regions with decreased metabolism. A serial PET study with longitudinal follow-up at multiple time points after surgical therapy highlighted the usefulness of CCD as an indicator of clinical outcomes [38]. Our data indicated that the phenomenon of CCD may play a pivotal role in treatment selection in steno-occlusive artery disease.

Decreased blood flow and energy failure are the main pathophysiologies of cerebral ischemia [39, 40]. Previous studies have investigated the relationship between perfusion and glucose metabolism using separate PET and MR procedures [41, 42]. The advantage of our approach is that we avoided functional and physiological variations using different modalities. Using hybrid PET/MR, we focused on the CCD-related supratentorial blood flow and metabolic status before bypass surgery. We previously found that the areas with reduced blood flow and the areas with reduced metabolism are not completely overlapping. We defined the co-decreased region in terms of both decreased CBF values and lower ^18^F-FDG uptake levels. The present study found there was a larger volume and a higher metabolism AI value in the CCD + group. The preprocedural clinical score had strong correlations with the metabolism AI value and volume of the co-decreased region. Although there was no statistical significance, the CBF AI value was slightly higher in the CCD + group. Paschen et al.[43] found that the compensatory increase of glucose metabolism at rCBF between 20 and 35 ml/100 g/min was sharply reduced at rCBF <20 ml/100 g/min in an animal model of ischemia. Based on the mechanism of stroke, we propose that the co-decreased region may have a higher risk of infarction. Bypass surgery may reduce risks in patients with CCD-related supratentorial blood flow and metabolic status.

The present study also had some limitations. First, the study was retrospective, which did not allow a sample size estimation, but we expect that our results will be confirmed in larger prospective study cohorts. Second, multiple PLDs should have been chosen. Absolute quantitative parameters were not measured using dynamic ^18^F-FDG PET on an integrated PET/MR system. Another limitation of this study was the lack of cerebellar quantification. Additionally, long-term PET/MR follow-up can help to monitor longitudinal hemodynamic and metabolic changes, which should be included in future prospective studies.

## Conclusions

In summary, the results of our study suggest that cerebral hemodynamic and metabolic patterns are important indicators of CCD. CCD + patients with chronic ischemic cerebrovascular disease received a significant benefit from bypass surgery. Staging based on the CCD-related supratentorial blood flow and metabolic status by hybrid PET/MR may help to personalize treatment.

## Supplementary Information

Below is the link to the electronic supplementary material.

Supplementary file1 (DOCX 444 KB)
