# Supplementary material for: Crossed cerebellar diaschisis-related supratentorial hemodynamic and metabolic status measured by PET/MR in assessing postoperative prognosis in chronic ischemic cerebrovascular disease patients with bypass surgery
Source: Ann Nucl Med. 2022 Jul 5;36(9):812–22. doi: 10.1007/s12149-022-01766-0 (PMC9374607; doi:10.1007/s12149-022-01766-0)
Supplement: Supplementary file 1 — Supplementary file1 (DOCX 444 KB) [file 12149_2022_1766_MOESM1_ESM.docx]

**Crossed cerebellar diaschisis****-related supratentorial** **hemodynamic and metabolic status measured by PET/MR in assessing postoperative prognosis in chronic ischemic cerebrovascular disease patients with bypass surgery**

Journal name: Annals of Nuclear Medicine

Bixiao Cui^1,2^, Yi Shan^1,2^, Tianhao Zhang^3,4^, Yan Ma^5^, Bin Yang^5^, Hongwei Yang^1,2^, Liqun Jiao^5^, Baoci Shan^3,4,6^, Jie Lu^1,2^

^1^Department of Radiology and Nuclear Medicine, Xuanwu Hospital, Capital Medical University, Beijing, China

^2^Beijing Key Laboratory of Magnetic Resonance Imaging and Brain Informatics, Beijing, China

^3^Institute of High Energy Physics, Beijing Engineering Research Center of Radiographic Techniques and Equipment, Chinese Academy of Sciences, Beijing, China

^4^School of Nuclear Science and Technology, University of Chinese Academy of Sciences, Beijing, China

^5^Department of Neurosurgery, Xuanwu Hospital, Capital Medical University, Beijing, China

^6^CAS Center for Excellence in Brain Science and Intelligence Technology, Shanghai, China

Bixiao Cui and Yi Shan contributed equally to this work.

**Corresponding author**

Jie Lu

Department of Radiology and Nuclear Medicine, Xuanwu Hospital, Capital Medical University, Beijing, China

Telephone: +86-18911507070

E-mail: imaginglu@hotmail.com

Fax number: 010- 83198376

**Supplementary files**

**Additional file 1.**


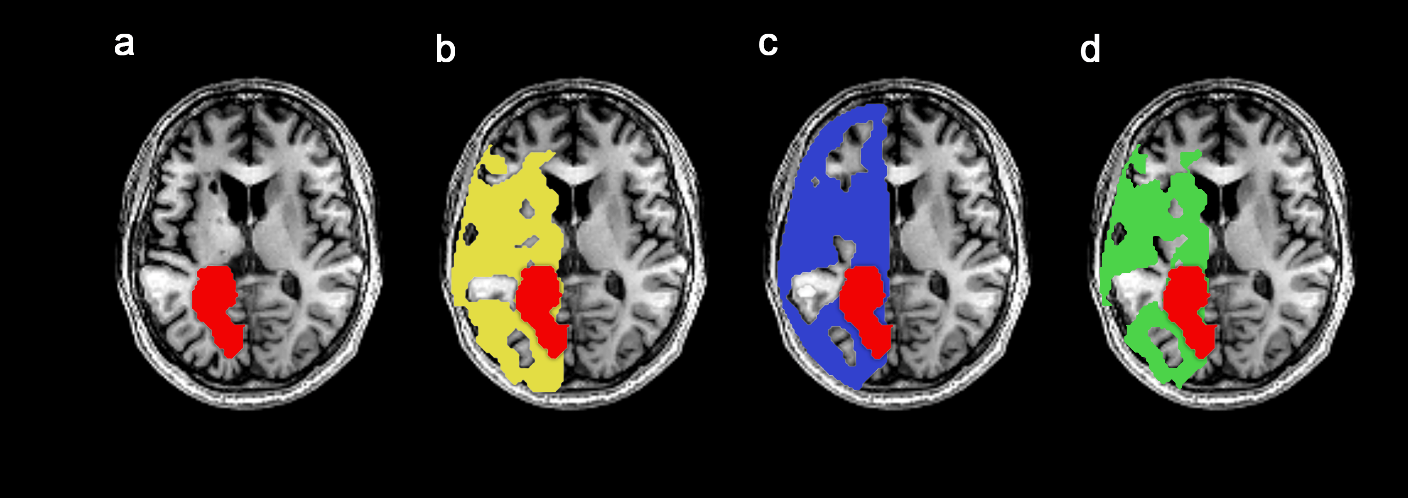


**Supplemental Fig. S1 Diagrams showing ROIs in a 3-dimensional stereotaxic ROI template.** Red areas indicate the infarct zone (a). Yellow and blue regions indicate regions with decreased CBF (b) and regions with decreased SUVR (c), respectively. The common regions where CBF and SUVR both decreased are shown in green (d).
